# Intersection of Brain Development and Paediatric Diffuse Midline Gliomas: Potential Role of Microenvironment in Tumour Growth

**DOI:** 10.3390/brainsci8110200

**Published:** 2018-11-16

**Authors:** Katie F. Loveson, Helen L. Fillmore

**Affiliations:** Brain Tumour Research Centre, University of Portsmouth, School of Pharmacy & Biomedical Sciences, PO1 2DT Portsmouth, UK; Katie.loveson@port.ac.uk

**Keywords:** DIPG, development, brainstem, tumour microenvironment

## Abstract

Diffuse intrinsic pontine glioma (DIPG) is a devastating and incurable paediatric brain tumour with a median overall survival of 9 months. Until recently, DIPGs were treated similarly to adult gliomas, but due to the advancement in molecular and imaging technologies, our understanding of these tumours has increased dramatically. While extensive research is being undertaken to determine the function of the molecular aberrations in DIPG, there are significant gaps in understanding the biology and the influence of the tumour microenvironment on DIPG growth, specifically in regards to the developing pons. The precise orchestration and co-ordination of the development of the brain, the most complex organ in the body, is still not fully understood. Herein, we present a brief overview of brainstem development, discuss the developing microenvironment in terms of DIPG growth, and provide a basis for the need for studies focused on bridging pontine development and DIPG microenvironment. Conducting investigations in the context of a developing brain will lead to a better understanding of the role of the tumour microenvironment and will help lead to identification of drivers of tumour growth and therapeutic resistance.

## 1. Introduction

Brain tumours are the leading cause of cancer-related death in children. Tumours of the central nervous system (CNS) are the second most common malignancy in children, after leukaemia [1,2,3,4]. Diffuse intrinsic pontine gliomas (DIPG, now reclassified as diffuse midline glioma), represent approximately 10% of childhood brain cancer, with medulloblastoma being the most common (20%) [5,6]. DIPG is a rare, devastating and incurable cancer with a median overall survival of 9 months with nearly all patients succumbing to this cancer within 2 years of diagnosis, with less than 1% surviving after 5 years. DIPG is considered an orphan disease with a yearly incidence of 2.32 per 1,000,000 people aged 0–20 years [3,7,8,9,10,11]. These tumours, which are restricted to the midline structures of the brain, primarily affect young children with peak incidence at 6 years of age. These have the highest mortality of all childhood solid tumours [10,12]. Since the brainstem controls basic life functions, surgical removal is not an option (except for biopsy), and chemotherapy and radiation only provide palliative relief [7,8,12]. The midline location and diffusive nature of the tumour causes severe disabling neurologic symptoms that over time destroy facial control and motor co-ordination. Due to the rapid progression of DIPG, children normally experience symptoms for a month or less before diagnosis [13,14]. The compression or dysfunctions of anatomic structures cause children to present with different clinical signs and symptoms, dependent on the tumour location. The increase in intracranial pressure can cause headaches, nausea, vomiting, and vision loss. Common clinical presentations of tumours located in the posterior fossa are ataxia and clumsiness [15]. The “classic triad” in DIPG patients are (1) cerebellar signs, e.g., ataxia dysmetria, and dysarthria; (2) long tract signs: weakness, spasticity, sensory loss, and abnormal reflexes; and (3) multiple cranial neuropathies, although only up to 50% of children may present with these [13,15,16,17]. Growth of DIPGs occur in a relatively discrete spatial and temporal pattern, which coincides with periods of developmental myelination suggesting a dysregulation of the postnatal neurodevelopmental process.

Previously, DIPGs were treated in the same way as adult gliomas, but recent advances in stereotactic neurosurgery for obtaining biopsy tissue for molecular analyses has led to the expansion of knowledge, notably the identification of somatic histone mutations [10,18,19]. Mutations in *H3F3A* or *HIST3BHI*, which encode the histone 3.3 (H3.3) and histone 3.1 (H3.1), respectively, lead to a substitution of methionine for lysine at position 27 (K27M). These appear to be present in 80% of DIPG patients, with tumours that arise throughout the midline structures harbouring the H3.3 K27M mutation, while H3.1 K27M are restricted to the pons [16,18,19,20,21,22,23]. Since the identification of this mutation, many studies have tried to understand the role of the histone H3K27M mutation in DIPG oncogenesis. These studies have shown that this mutation suppresses activity of enhancer of zeste 2 (EZH2), the catalytic sub-unit of polycomb repressive complex 2 (PRC2), causing a marked reduction in H3K27 methylation, resulting in a re-wiring of an essential developmental regulator of genes [10,12,24,25,26,27,28,29]. These histone mutations modify the epigenome, causing oncogenic insults to progenitor cells in early neurodevelopment [10,30,31]. It is suggested that these histone mutations may contribute to an initial oncogenic event; however, are not solely sufficient for the formation of the tumour and are associated with other gene mutations such as cell cycle regulators (*TP53, PPMID*), the chromatin remodeler (*ATRX*), or growth factors (*ACVR1, PDGFRA*) [18,19,22,32,33,34].

In addition to molecular analysis, tissue collections have enabled the establishment of primary tumour cell lines and patient-derived xenograft (PDX) models to further understand the biology of the tumour. Lagging behind are studies designed to understand oncogenic events in the context of the tumour microenvironment [35,36,37,38]. Unfortunately, DIPG is still not well understood, partly because of its low incidence, barrier to tissue acquisition (biopsy), and autopsy. Additionally, the molecular characterisation has not yet been translated into better treatments [39,40]. Connections with neural development and diffuse gliomas are suggested by the markedly different neuroanatomical locations across different age groups. For example, tumours with H3.1 K27M are restricted to the pons and are found in younger children, whereas the H3.3 K27M mutation is present in tumours located in any midline structure and tends to occur in older children [2,10,41]. Herein, we present a brief overview of brainstem development, discuss the developing microenvironment in terms of DIPG growth, and provide rationale for the need for more knowledge into pontine development and the DIPG microenvironment. 

## 2. Development

Brain development takes over two decades via precisely regulated molecular, cellular, and epigenetic processes that are governed by a genetic blueprint and environmental factors. When this process is interrupted, pathologies arise. Early brain development, particularly from the mid-foetal stage to 2 years after birth, is the most dynamic across the entire lifespan [42]. The cellular and environmental composition of the paediatric brain is very different to that of the adult brain and consists of a large number of proliferating, migrating, and differentiating cells. The mature brain is comprised of approximately 86.1 billion neurons, as well as equal numbers of glial cells (oligodendrocytes and astrocytes) [43,44,45,46]. While much is known about gliogenesis in cortical areas, we continue to gain knowledge relating to less studied regions of the human brain from genomic data (brainspan.org) and from advances in imaging technology incorporating biophysical models in which early developmental changes are being mapped [42,47,48,49,50,51,52].

### 2.1. Brainstem

The brainstem includes the midbrain, the pons, and the medulla oblongata. The pons not only serves as a bridge between the cerebrum and the spinal cord; it is also home to many cranial nerve ganglia that are involved in the co-ordination of motor control signals sent from the brain to the body. This area of the brain is responsible for the control of several important functions of the body including alertness, arousal, breathing, blood pressure, digestion, heart rate, swallowing, walking, and sensory and motor information integration [53,54].

**Figure 1:**
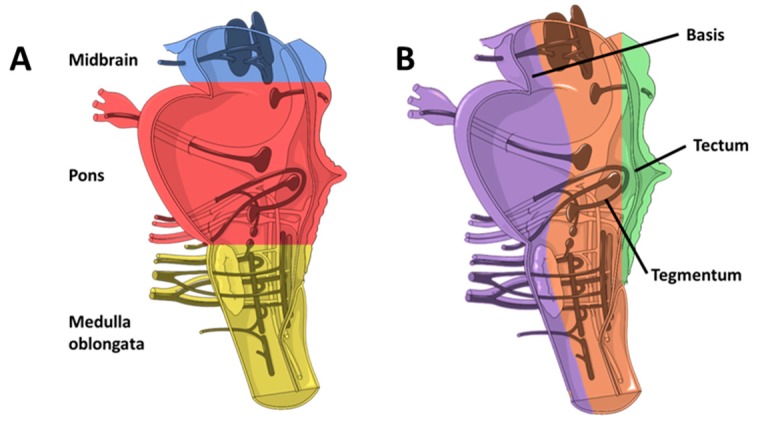
Anatomical structures of the brainstem showing cranial nerves. (**A**) The brainstem is divided into the Midbrain (blue), Pons (red), and the Medulla Oblongata (yellow). (**B**) Laminae of the brainstem: Basis (purple), Tegmentum (brown), and Tectum (green). Schematic modified from [54].

The brainstem develops from two of three primary regions formed from the neural tube. The three regions are forebrain, midbrain, and hindbrain. The forebrain ultimately becomes the cerebrum and the diencephalon. The midbrain region or mesencephalon becomes the midbrain in adults. The hindbrain develops into two regions; the metencephalon, which will form the pons and the cerebellum and the myelencephalon, which will become the medulla oblongata [47,55,56]. The pons is located between the midbrain and the medulla oblongata, with a presenting anterior surface connecting the right and left cerebellar hemispheres [54] (Figure 1A). The brainstem contains both white matter and grey matter. Grey matter (collection of neuronal cell bodies) is found throughout the brainstem and includes cranial nerve nuclei (10 of the 12 cranial nerves, III to XII), the reticular and pontine nuclei. The white matter consists of fibre tracts passing down from the cerebral cortex to the cerebellum and spinal cord and up from the peripheral nerves and the spinal cord [54]. The internal structure of the brainstem is organised into three laminae: basis (ventral), tectum (dorsal), and tegmentum (medial), which extend the length of the brainstem [54] (Figure 1B).

There are three phases of growth in the basis of the pons. In the first three months of life, the basis dramatically expands in volume as compared to the tegmentum because during the first month, the proliferation is increased in the basis relative to the tegmentum. Between 3 months and 1 year, this expansion decreases. After 1 year, the growth rate of the basis declines to near zero until 7 years. In contrast, the growth rate of medulla and tegmentum are dramatically reduced by 3–6 months and 6 months-1 year, respectively [47,57].

Although not intended to be an exhaustive review on pontine nuclei, their cortical input, and their projections, there are a few highlights we present for this review and refer to several outstanding detailed reviews on this subject [54,58,59]. Pontine neurons are derived from the neurodevelopmental anatomical area referred to as the rhombic lip, and these neurons migrate in several phases during development. Depending on the origination of the specific rhombmere these neurons migrate a long distance in one direction and change at specific sites. This tangential or rostroventral migration of pontine neurons along the anterior extramural stream or posterior extramural stream is well orchestrated and dependent on developmental regulated mechanisms including transcription factors and attractive and repulsive proteins [54]. The pontine nuceli (PN) receive most of their input from the cortex and this afferent connection is called the cortical-pontine projection and has a specific topography associated with it [59]. The pontine-cerebellar is an efferent pathway with PN projections terminating in the cerebellum. 

Developmentally and during the median age of diagnosis of DIPG, the pontine region is a very busy area with migration of neurons to specific brainstem nuclei, formation of synaptic input from the cortex, and subsequent projections to the cerebellum and cortico-spinal (ascending and descending) pathways to name a few multifaceted processes. A vital key and an essential post-natal event involves the myelination of the above mentioned pathways. Myelination is vital for the successful establishment of efficient brain communication. 

### 2.2. Myelination

Myelination involves a step-wise process that requires oligodendrocyte progenitor generation and proliferation; cell migration and differentiation into oligodendrocytes; process extension and their interaction with axons; synthesis and trafficking of membrane, wrapping, and compaction; and the establishment of axo-glial junctions [60,61,62]. The coating of the axon of each neuron with a lipid-rich coating called myelin is essential for normal brain function and is a cornerstone of human neurodevelopment [63,64]. The myelin sheath, a multi-lamellar, lipid-rich structure is essential for rapid propagation of action potentials, but also protects neuronal axons. This is one of the most pivotal cell-cell interactions for normal brain development, allowing extensive exchange of information between mature oligodendrocytes and axons [60,64,65]. While progress to identify the molecular and cellular mechanisms of myelination, as well as identification of the individual molecules involved have been made, there is still a lack of understanding of the communication required [60,66]. Myelination begins in the brain stem and cerebellum before birth and continues through childhood, but is not completed in the frontal cortex until late in adolescence [64,67]. Studies using rodent models have limitations as myelination begins during neurogenesis and only takes a few weeks in comparison to decades in humans [68].

**Figure 2:**
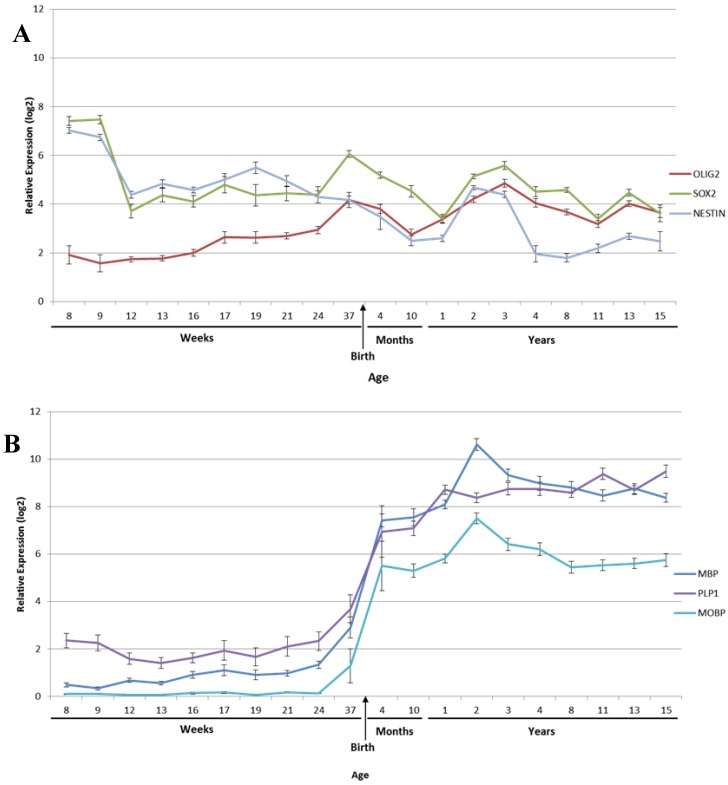
Timeline of genes identified in the central nervous system (CNS) development throughout development from conception to 15 years of age. Data were obtained from brainspan.org and analysed using R2. (**A**) OLIG2, SOX2, and Nestin positive cells have been identified as the main proliferating cells in early postnatal brainstem development, with a decrease around 3 years of age. (**B**) Myelinating genes show a rapid increase in expression before birth until 2 years of age, before plateauing. (Myelin Basic Protein, MBP, Proteolipid Protein, PLP1, Myelin-Associated Oligodendrocyte Basic Protein, MOBP).

Despite the critical functions of the brainstem, postnatal development is still poorly understood, with the brainstem frequently ignored in brain development studies or studies describing the development of the foetal pons [43,57,69,70]. A recent magnetic resonance imaging (MRI) study of human specimens reveals that the pons increases 6-fold in size from birth to 5 years of age, with continued slower growth throughout childhood [47]. In the first month after birth, neural progenitor cells (NPCs) rapidly proliferate, then gradually decline until 7 months of age. Proliferating NPCs in both the human and rodent developing pons have been shown to express Nestin (intermediate NPC filament), SOX2 (stem/progenitor cell transcription factors sex determining region Y-box 2), and OLIG2 (basic helix-loop-helix transcription factor) in a co-expressing and independent manner [36,47,71]. Monje et al, examined the spatial and temporal distribution of neural precursor cells in postnatal human brainstems, observing that the majority of the proliferating cells in the pons were OLIG2^+^ [36]. This dramatic increase in size has also been observed in a mouse model, where the main proliferating cells expressed oligodendrocytic lineage markers SOX2 and OLIG2 and not the addition of new neurons [36,71,72], suggesting the increased myelination is due to the development of neural circuits through learned behaviour during childhood development [10,47,73,74] (Figure 2).

### 2.3. Oligodendrocytes

Oligodendrogenesis, the generation of mature, myelinating oligodendrocytes involves a precise balance with epigenetic regulation of differentiation activators and inhibitors, followed by the transcriptional activation of myelin genes [75,76,77]. Oligodendrocytes (from Greek meaning ‘cells with a few branches’) are responsible for structural support and the formation of the myelin sheath around the axons to ensure rapid impulse propagation [68]. 

**Figure 3:**
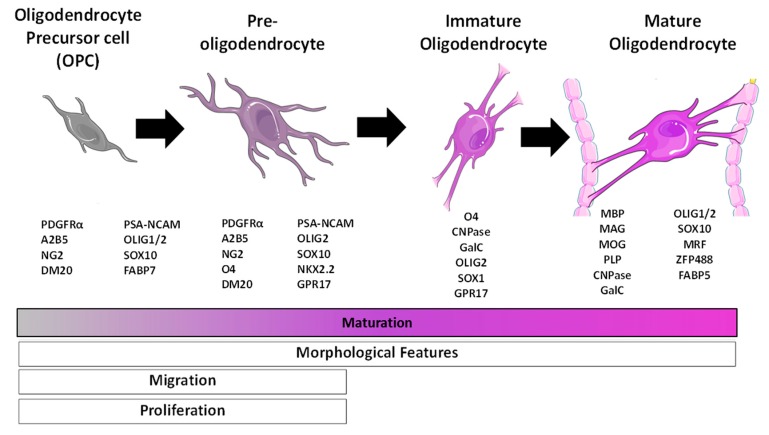
Specific proteins and transcription factors are expressed at the different stages of oligodendrocyte (OL) differentiation. These stages are recognised by the expression of well-characterised markers, distinct morphology, and their ability to proliferate, migrate, and differentiate. Modified from [79].

Oligodendrocytes arise from oligodendrocyte precursor cells (OPCs) in a step-wise process that involves specification, proliferation, and differentiation requiring co-ordination of transcriptional and epigenetic circuits to mediate the stage-specific intricacies of oligodendrocyte development. This is driven by the interplay of extracellular signals, including secreted molecules, neuronal activity, extracellular matrix components, and spatial constraints in the microenvironment with the intracellular molecular components, such as transcription factors and epigenetic regulators [78]. The stages of oligodendrocyte maturation are divided into four different steps: oligodendrocyte precursor cells (OPCs), pre-oligodendrocytes (or late OPCs), immature (or pre-myelinating) oligodendrocytes (OLs), and mature (or myelinating) OLs [79]. These stages are well described and are identified by expression of specific proteins and transcription factors [80,81,82,83] (Figure 3). An extensive description of these are beyond the scope of this review and interested readers are directed to a number of excellent reviews elsewhere [79,82,84,85]. 

It is still unclear whether the OPCs receive multiple negative signals acting in parallel or they all come together in one signalling pathway, but they appear to have growth cone-like structure, which they appear to use to explore their environment [86].

**Figure 4:**
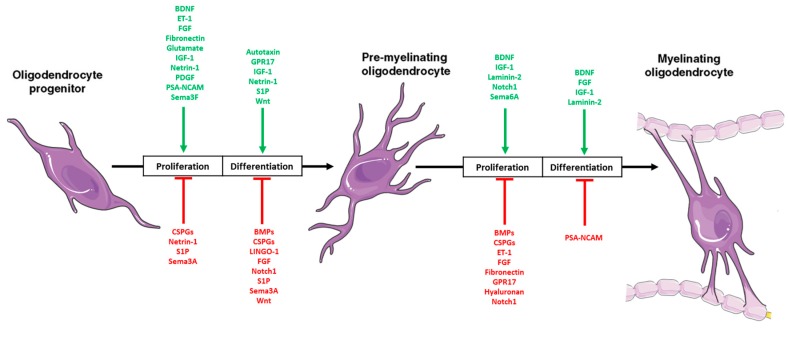
Schematic representation of the generation of mature, myelinating oligodendrocytes. A precise balance with epigenetic regulation of differentiation activators and inhibitors of extracellular cues promoting (green) and/or inhibiting proliferation (red) and/or differentiation at different stages of oligodendrogenesis. Modified from [88].

OPCs also exhibit predominantly euchromatic nuclei, defined by a relaxed chromatin structure and easy DNA accessibility, potentially making them more susceptible to mutations [78,87]. These progenitor cells exhibit greater proliferation and tumour propagating potential than their more differentiated counterparts. OPCs proliferation is actively stimulated by extracellular signals, that not only promote proliferation but also inhibit differentiation, for example, platelet-derived growth factor (PDGF) has been shown to potently drive OPC proliferation [67,81] (Figure 4).

### 2.4. The Developing Microenvironment: Intersection with DIPG Microenvironment

The development of the brain requires a co-ordination of molecular and cellular processes across an array of cell types over a period of time [43]. The precise choreography of numerous components of the developing microenvironment is necessary for successful connections and the ultimate functioning of neural networks. CNS development involves a highly constrained, dynamic, and organised process of stem cell self-renewal and differentiation determined by both genetic and environmental factors in an orderly pattern [89,90]. 

**Figure 5:**
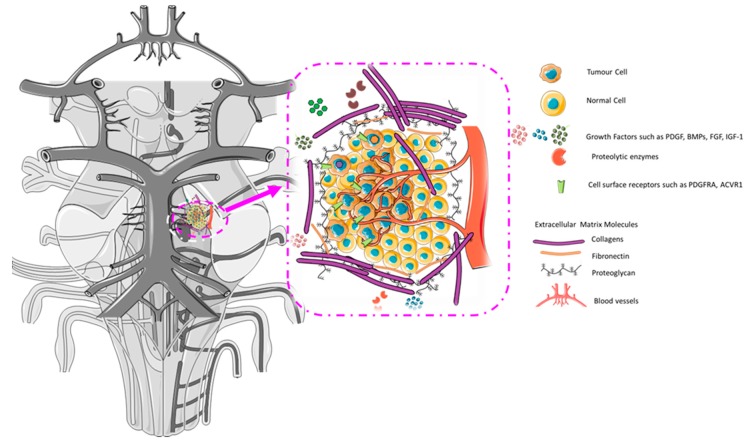
Schematic representation of the tumour microenvironment of a diffuse intrinsic pontine glioma (DIPG) in the context of the brainstem. Paediatric brain tumours form in the context of the developing CNS, adding an extra layer of complexity compared to adult brain tumours. By understanding the signalling pathways that govern brainstem development and the way they interact with the tumour, we can try to dissect the drivers of growth and resistance. Not depicted are microglia, immune components, and neural satellitosis.

The tumour microenvironment (TME) is a complex regulatory and dynamic structure composed of cellular and non-cellular components and processes that contribute to disease progression. There are a wide range of physiological mechanisms that can fall under TME, spanning from metabolism to biomechanical processes. TME is being increasingly recognised as a key factor in multiple stages of cancer progression, particularly in regards to local resistance, immune-escaping, and promoting distant metastasis [91]. Brain tumour cells are part of a dynamic and spatially distributed system, interacting with a wide diversity of environments and cell types [92]. In addition to cancer cells, tumour lesions contain a mixture of different stromal cells such as endothelial cells (EC) and inflammatory cells that infiltrate tumours (Figure 5). 

New information concerning the immune landscape is beginning to be realised with current reports demonstrating that DIPG exhibits a less inflammatory microenvironment compared to that of adult high grade gliomas [93,94]. These studies, in addition to the outcomes of several immunotherapy based clinical trials, will help inform future trials [95]. Nevertheless, there is still a need to improve our basic understanding of the developing brainstem in all aspects including microglial dynamics [96]. Information on the spatial and temporal expression of genes and proteins involved in modulating the immune system during development will be vital when considering immunotherapy strategies.

Tumour cells proliferate, remodel, attach, and rebuild a new microenvironment by releasing extracellular signalling molecules that promote tumour angiogenesis, extracellular matrix (ECM) remodelling, and evasion of the immune system [97]. The cancer cells exploit the bidirectional communication between healthy glial cells, endothelial cells, and neurons to remodel the microenvironment to grow and evade therapeutics.

Whilst extensive research is being undertaken to determine the function of molecular aberrations in DIPG, there are significant gaps in understanding the influence of the tumour microenvironment and the development of the pontine area of the brain stem [25,26,98]. Additional developmental processes that need addressing include the formation of the various brain barriers such as the blood brain barrier, blood-cerebrospinal fluid (CSF), and arachnoid barrier.

The brain is one of the most densely vascularised organs; the blood vessels differ from the blood vessels in other organs in terms of their tightness and structure. Tight junctions between brain EC and metabolic barriers strongly resist the passage of cells and even small molecules through the blood-brain barrier (BBB). Furthermore, blood vessels are supported by astrocyte end feet and pericytes [99]. In adult glioma, a ‘leaky blood brain barrier’ is well described [100], whereas there is limited descriptive clinical data in DIPG. The use of MRI enhancement suggests that DIPG tumours at diagnosis have a relatively intact and functional BBB [101,102]. Recent data from a non-human primate model suggests that the pons has a super-BBB compared to the cortex, further restricting substances to the brain [100,103].

Two thirds of the CSF is produced from the choroid plexus, structures located within the lateral, third, and fourth ventricles. The development of the choroid plexus is interesting, and although much is known concerning the development, less is known about these structures in DIPG. Could the choroid plexus contribute in some way to the dissemination of tumour cells? Are there changes associated with the choroid plexus in patients diagnosed with DIPG? During development, arteries invaginate the roof of ventricles to eventually form involuted ependymal cells containing connective tissue and many fenestrated blood vessels and become the blood-CSF barrier [104,105]. This is a well-studied area in neural development and to cover the exciting work that has led to the characterisation and identification of factors involved in the establishment of a functioning blood-CSF barrier is beyond the scope of this review and so we refer readers to another excellent review [104]. The purpose of mentioning these barriers is that they are part of the DIPG microenvironment and may at some level affect, associate with, or participate in DIPG growth. These barriers are also important for the protection of the brain as well as providing essential nutrients. In addition, learning more about these structures and their functions in normal development as well as in DIPG could potentially provide information on delivery routes for therapies [106,107].

How the developing brain microenvironment contributes or interferes with DIPG is unknown, and it most likely is multifaceted and dynamic both in space and time. The task is to distinguish what are normal developmental processes and what are DIPG related. Clues can be obtained from numerous studies into identification of the tumour cell of origin.

## 3. Cell of Origin

The term ‘cell-of-origin’ refers to the normal cell type that is uniquely susceptible to particular oncogenic mutation(s) resulting in a tumour [108]. Glial cells have a prominent role in the development and physiology of the brain. The word glioma comes from their similarity, morphologically, to the normal glial cells of the brain. The cell of origin for DIPG is not known [10,36], but recent data suggests an oligodendrocytic lineage cell [10,12,36,90,108,109,110]. This is supported by the expression of essential factors in the specification of oligodendrocytes, *PDGFRA* amplification, chondroitin sulfate proteoglycan NG2, and basic helix-loop-helix transcription factors: OLIG1 and OLIG2, are up-regulated in 80% of DIPG cases [1,71,111,112]. It has been suggested that DIPG may arise from an aborted cell differentiation program of the developing pons, resulting in uncontrolled proliferation [40]. Interestingly, there may be a distinct difference in the cell of origin of these tumours as it has been shown that the H3.3 K27M mutated DIPG have a proneural/oligodendroglial phenotype with a pro-metastatic gene expression signature with *PDGFRA* activation, while H3.1 K27M mutated tumours exhibit a mesenchymal/astrocytic phenotypic phenotype [113]. It has been suggested that the gene expression signature may not be due to the specific histone mutation, but rather the accompanying alterations (*PDGFRA* vs. *ACVR1*), supporting the notion that modulation of the microenvironment by tumour cells is more influential than the histone mutation [10,113].

## 4. Conclusions

While extensive research is being undertaken to determine the function of the molecular aberrations in DIPG, there are significant gaps in understanding the biology and the influence of the tumour microenvironment on DIPG growth, specifically in consideration of the developing pons. The precise orchestration and co-ordination of the development of the brain is still not fully understood. Cancers do not grow on their own, and we believe that knowing more about the microenvironment in the developing CNS is critical for understanding the drivers of tumour growth and therapeutic resistance in DIPG. The intersection of brain development and paediatric brain cancer in terms of microenvironment holds critical information for a major shift in the ways these tumours are treated.
